# Midfrontal theta as an index of conflict strength in approach–approach *vs* avoidance–avoidance conflicts

**DOI:** 10.1093/scan/nsad038

**Published:** 2023-07-24

**Authors:** Ariel Levy, Maya Enisman, Anat Perry, Tali Kleiman

**Affiliations:** Department of Cognitive and Brain Sciences, The Hebrew University of Jerusalem, Mt. Scopus, Jerusalem 9190501, Israel; Department of Psychology, The Hebrew University of Jerusalem, Mt. Scopus, Jerusalem 9190501, Israel; Department of Psychology, The Hebrew University of Jerusalem, Mt. Scopus, Jerusalem 9190501, Israel; Department of Psychology, The Hebrew University of Jerusalem, Mt. Scopus, Jerusalem 9190501, Israel

**Keywords:** midfrontal theta, motivational conflicts, approach, avoidance, cognitive control

## Abstract

The seminal theory of motivational conflicts distinguishes between approach–approach (AP-AP) conflicts, in which a decision is made between desirable alternatives, and avoidance–avoidance (AV-AV) conflicts, in which a decision is made between undesirable alternatives. The behavioral differences between AP-AP and AV-AV conflicts are well documented: abundant research showed that AV-AV conflicts are more difficult to resolve than AP-AP ones. However, there is little to no research looking into the neural underpinnings of the differences between the two conflict types. Here, we show that midfrontal theta, an established neural marker of conflict, distinguished between the two conflict types such that midfrontal theta power was higher in AV-AV conflicts than in AP-AP conflicts. We further demonstrate that higher midfrontal theta power was associated with shorter decision times on a single-trial basis, indicating that midfrontal theta played a role in promoting successful controlled behavior. Taken together, our results show that AP-AP and AV-AV conflicts are distinguishable on the neural level. The implications of these results go beyond motivational conflicts, as they establish midfrontal theta as a measure of the continuous degree of conflict in subjective decisions.

## Introduction

Would you rather be rich or smart? Poor or stupid? Lewin’s (1931) and Miller’s (1944) seminal motivational conflict theory distinguishes between two types of conflicts: deciding between being rich and smart (Arkoff, 1957) is an example of an approach–approach (AP-AP) conflict, in which the decision is made between desirable alternatives, whereas deciding between being poor and stupid is an example of an avoidance–avoidance (AV-AV) conflict, in which the decision is made between undesirable alternatives. Both Lewin’s and Miller’s original conceptualizations, as well as abundant empirical research that followed, predicted and showed that AV-AV decisions elicit greater conflict (i.e. are more difficult to resolve) than AP-AP ones. Specifically, participants find AV-AV conflicts to be more difficult to resolve than AP-AP conflicts and take substantially longer to resolve them (Arkoff, 1957; Barker, 1946; Atthowe, 1960; Minor *et al.*, 1968; Murray, 1975; Chatterjee and Heath, 1996; Terry, 2010; Boyd *et al.*, 2011; Perfecto *et al.*, 2017; Heitmann and Deutsch, 2019). The behavioral differences between AP-AP and AV-AV conflicts are thus well documented. However, there is little to no research to date looking into the neural underpinnings of the differences between the two motivational conflict types. In the present paper, we draw from the vast body of research on the neural mechanisms that underlie the processing of conflicts (e.g. Botvinick *et al.*, 2001; Cavanagh and Frank, 2014; Cohen, 2014; Larson *et al.*, 2014) to gain insight into how motivational conflicts unfold.

The question of how conflicts—‘the simultaneous activation of incompatible, and competing, representations’ (Botvinick *et al.*, 2004, p. 541)—are monitored, processed and resolved in the brain has received abundant research attention (MacDonald *et al.*, 2000; Botvinick *et al.*, 2001; Vanveen and Carter, 2002; Carter and van Veen, 2007; Cavanagh and Frank, 2014; Cohen, 2014). To date, most of the research regarding the neural underpinnings of conflict has focused on conflicts induced by stimulus-response compatibility (SRC) tasks (e.g. Kornblum *et al.*, 1990; Egner, 2008), such as the Eriksen Flanker task (Eriksen and Eriksen, 1974) or the Stroop task (Stroop, 1935). In SRC tasks, conflict trials and no-conflict trials are created by constructing stimuli in which the target and distractor elicit either incompatible responses (e.g. the word RED written in blue ink in the Stroop task) or compatible ones (e.g. the word RED written in red ink), respectively. This line of research identified two central electroencephalogram (EEG)-based approaches to study conflicts. First, event-related potentials (ERPs), such as the N2 (Vanveen and Carter, 2002), conflict-related negativity (CRN; Nakao *et al.*, 2013) and the lateral positive complex (LPC, also known as conflict slow potentials; Appelbaum *et al.*, 2009; Larson *et al.*, 2012; McKay *et al.*, 2017), are thought to reflect different aspects of conflict processing (see Larson *et al.*, 2014, for a review). Second, midfrontal theta (∼4–8 Hz) oscillations are considered a robust neural marker of conflict processing and of recruitment of control needed to resolve the conflict (Cavanagh *et al.*, 2012; Cohen and Donner, 2013; Frank *et al.*, 2015; Hsieh *et al.*, 2020; Kaiser *et al.*, 2022).

Both SRC conflicts and motivational conflicts are conflicts with respect to the inherent characteristic of conflict. In both cases, one needs to decide (i.e. resolve the competition) between two incompatible alternatives. However, SRC conflicts and motivational conflicts also differ in several ways. First, in SRC tasks, there are either conflict trials or no-conflict trials, and so conflict is arguably manipulated in a ‘discrete’ manner. In motivational conflicts, both AP-AP and AV-AV decisions elicit conflict. The difference is thus in the degree of conflict, which is ‘continuous’ rather than discrete. Second, SRC conflicts typically take a few hundred milliseconds to resolve, whereas motivational conflicts typically take several seconds to resolve (Arkoff, 1957; Minor *et al.*, 1968; Terry, 2010; Heitmann and Deutsch, 2019). Third, in SRC conflicts, there is an ‘objectively correct response’ (answer) set by the task rules (e.g. responding ‘blue’ to the word RED written in blue ink). Motivational conflicts, in contrast, are based on a ‘subjective’ structure of preference, and so there is no objectively correct or incorrect decision.

Considering these differences is important when making predictions regarding which of the two central neural markers of conflict (ERPs or theta oscillations) will be most suitable in capturing the difference between AP-AP and AV-AV conflicts. ERPs seem like less suitable candidates. To date, conflict-related ERPs have mostly been obtained in tasks where there is an objectively correct response, in which response times (RTs) are rather short, and when comparing discrete conflict trials to no-conflict trials. Notable exceptions are studies that used a specific career choice paradigm and found a CRN effect for relatively long, subjective (Nakao *et al.*, 2010, 2013; Di Domenico *et al.*, 2013) and continuous (Di Domenico *et al.*, 2016) decisions, the latter being a fairly small effect (Di Domenico *et al.*, 2016, p. 860). However, other works using conflict tasks with relatively long decision times (Stahl *et al.*, 2020; Givon *et al.*, 2022) and involving subjective (Lin *et al.*, 2018; Givon *et al.*, 2022) and continuous (Lin *et al.*, 2018) decisions have not found conflict-related ERP effects.

Theta oscillations seem more likely to function as an index of conflict processing even when considering the unique characteristics of motivational conflicts described earlier. Two recent papers have looked at theta oscillations elicited by conflicts outside of the context of SRC tasks (Lin *et al.*, 2018; Senftleben and Scherbaum, 2021); theta was found to track conflict in both intertemporal (Lin *et al.*, 2018) and value-based decisions (Senftleben and Scherbaum, 2021), which are subjective and rather long (Lin *et al.*, 2018; Senftleben and Scherbaum, 2021), and which involve continuous degrees of conflict (Lin *et al.*, 2018). Taken together, these findings suggest that midfrontal theta oscillations can track long decisions and importantly are sensitive to the degree of subjective conflict and not only to the mere presence (*vs* absence) of conflict.

In the present research, we thus aimed to depict the neural underpinnings of the difference between AP-AP and AV-AV conflicts using midfrontal theta oscillations as the neural marker. Participants completed a motivational conflict task (modified from the classic paradigm of Arkoff, 1957), in which they repeatedly decided between two positive (e.g. to be smart or rich) or negative (e.g. to be stupid or poor) personal characteristics, constituting AP-AP and AV-AV conflicts, respectively. While completing the task, a continuous EEG signal was recorded. Conflict-related indices, including midfrontal theta and ERP components, were extracted from the EEG data and were used to examine the differences between the processing of AP-AP and AV-AV conflicts.

## Methods

### Participants and design

Thirty-two undergraduate students (75% female, range_age_ = 19–35, *M*_age_ = 24.7, s.d._age_ = 3.19) participated in the study for either course credit or the equivalent of ∼12 US dollar/h. We determined the sample size based on the robust and large behavioral effect of the resolution time difference between AP-AP and AV-AV conflicts obtained in previous studies (*d * > 1, Boyd *et al.*, 2011; Heitmann and Deutsch, 2019). Power analysis for the behavioral effect, with *d* = 1, *α* = 0.05 and 80% power, yielded a target sample size of 10 participants. We thus reasoned that tripling this number would be sufficient to detect the underlying neural underpinnings of this large behavioral effect. We manipulated conflict type: AP-AP *vs* AV-AV, in a within-participant design. The study was approved by the institutional review board.

### Motivational conflict experimental task

**Fig. 1.**
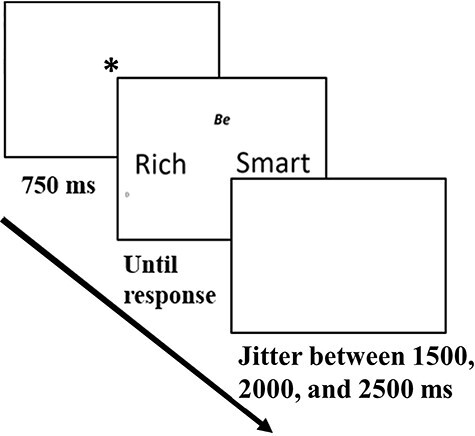
A depiction of an AP-AP conflict trial sequence in the motivational conflict task.

The task (a modification of the classic paradigm developed by Arkoff, 1957) was programmed using ‘Psychopy’ (Peirce *et al.*, 2019). We created AP-AP conflicts by pairing every combination of two characteristics out of seven positive personal characteristics (e.g. being ‘Smart’ or ‘Rich’) and AV-AV conflicts by pairing every combination of two characteristics out of seven negative personal characteristics (e.g. being ‘Stupid’ or ‘Poor’). This procedure resulted in a total of 21 conflicts for each of the motivational conflict types (see the Appendix for the full list of personal characteristics). An experimental block contained 42 randomly presented conflict trials of both types. The task consisted of seven experimental blocks, resulting in a total of 294 trials. At the beginning of each trial, an asterisk-shaped fixation point appeared at the center of the screen for 750 ms. Then, two personal characteristics (either positive or negative) appeared side by side in the middle of the screen. The location of a specific personal characteristic on either the left or the right side was determined randomly. The choice options remained onscreen until the participant pressed one of the two designated keyboard keys (‘D’ for the left option and ‘L’ for the right option). The intertrial interval jittered randomly between 1500, 2000 and 2500 ms (see Figure 1 for a depiction of the sequence of a trial).

### Procedure

After providing informed consent, participants read that this was a study on how people make decisions. They were told that there are no right or wrong answers and were asked to ‘Be sure that your decision is the one that you would actually make if you really had to decide’ (Arkoff, 1957). Following the instructions, participants completed four practice trials, two for each conflict type (in which we used different personal characteristics from the ones used in the experimental task) and then moved on to the seven experimental blocks. Between each of the blocks, there was a break that lasted until participants indicated that they were ready to proceed to the next block. Finally, participants provided demographic information, were asked whether they had any comments and debriefed. The experimental task took about 30 min to complete.

#### EEG recording

A continuous EEG signal was recorded using a BioSemi ActiveTwo recording cap with 64 active electrodes (BioSemi B.V., Amsterdam, The Netherlands), which were placed according to the international 10/20 system. All electrodes were referenced during recording to a common-mode signal electrode between POz and PO3 and were subsequently rereferenced digitally (see details later). Eye movements, as well as blinks, were monitored using bipolar horizontal and vertical electrooculography (EOG) derivations via two pairs of electrodes: a horizontal electrooculogram was recorded from electrodes located at the outer canthi of the right and left eyes and a vertical electrooculogram was recorded from electrodes located below and above the center of the right eye. Both EEG and EOG were digitally amplified and sampled at 2048 Hz.

### Data preprocessing

#### Behavioral data

The data of three participants who were excluded from the analysis based on the EEG exclusion criteria (see details later) were also excluded from the behavioral analysis,^1^ leaving the data of 29 participants for analysis. In addition, trials in which RT deviated ±3 s.d.s from the participant’s mean RT were excluded from the analysis (1.74% of the trials).

#### EEG data

All offline preprocessing stages were performed via the EEGLAB toolbox, an online open-source package for EEG processing (Delorme and Makeig, 2004) and the ERPLAB toolbox (version 8.001; Lopez-Calderon and Luck, 2014). EEG and EOG signals were downsampled to 512 Hz to increase data-processing speeds. Data were then high-pass-filtered (Butterworth filter with a half-amplitude cutoff at 0.1 Hz) to exclude slow drifts in the data. After we rereferenced the data to the average reference (including all 64 scalp electrodes), the EEG was subjected to an independent component analysis decomposition. Components that were clearly associated with eye movements were removed (Jung *et al.*, 2000).

**Fig. 2.**
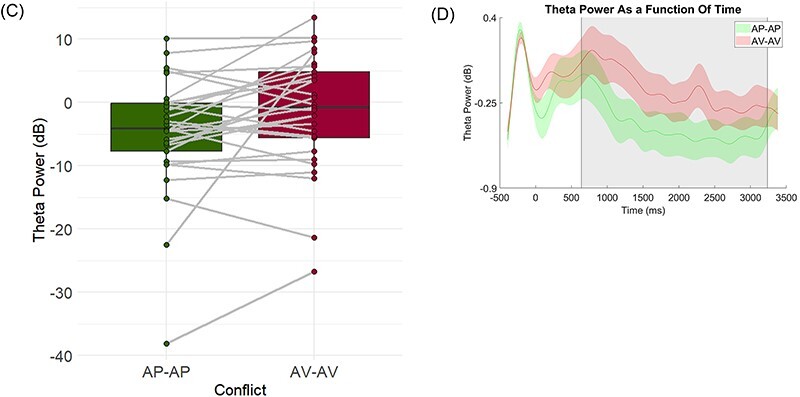
Time–frequency analysis results. (A) The time–frequency decomposition, aggregated by condition. (B) Topographical plots for theta power for each condition and for the average difference between conditions. (C) A box plot of overall midfrontal theta power as a function of conflict type, measured in a time window of 0–3000 ms following stimulus onset. The middle line represents the median and the whiskers represent 1.5 times the interquartile range. The pairs of connected dots represent the average of midfrontal theta power for each participant in each of the conflict conditions. (D) The averaged midfrontal theta power for AP-AP and AV-AV trials as a function of time from stimulus onset. The time window of the condition effect, as computed via the cluster permutations test, is marked in gray.

To create stimulus-locked ERPs, we extracted 2500 ms stimulus-locked epochs starting 500 ms before stimulus onset, which we baseline-corrected with a time window from −500 to 0. Channels with excessive levels of noise were interpolated using EEGLAB’s spherical interpolation algorithm (these channels were not used to quantify ERP components, and therefore, interpolation impacted only the topographic maps shown in Figures 2 and 4). Then, we performed artifact rejection on the stimulus-locked epochs, using the ‘moving window peak-to-peak’ method (Luck, 2014), with a window width of 100 ms and individual threshold parameters. We excluded the data of participants who exhibited artifacts in >25% of the trials from the analysis (Luck, 2014). Three participants met this exclusion criterion, and so we excluded their data from the analysis, leaving the data of 29 participants for the analysis. Out of the data of the 29 remaining participants, 17% of the trials were rejected in the artifact-rejection process.^2^ The remaining trials were averaged at the participant’s level to create ERPs, which were then low-pass-filtered, using a Butterworth filter with a half-amplitude cutoff at 30 Hz.

**Figure:**
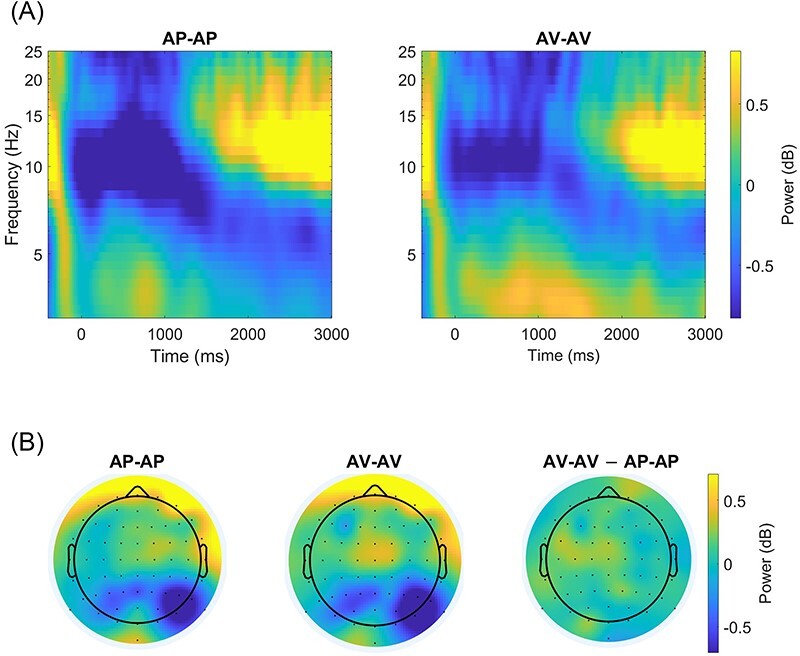


**Fig. 3.**
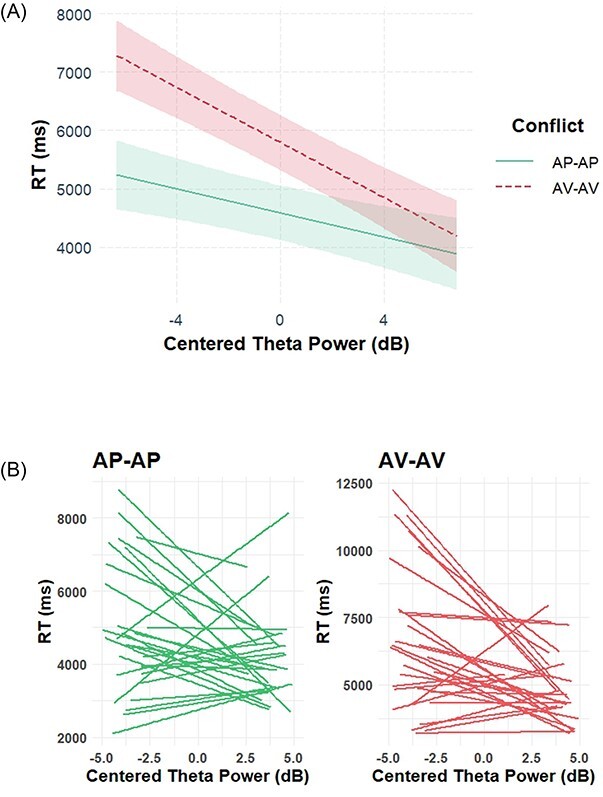
(A) Mixed-model predictions of RT as a function of theta power in AP-AP and AV-AV conflicts. (B) Individual regression lines for each participant, depicting the relationship between theta power and RT, in both conditions.

**Fig. 4.**
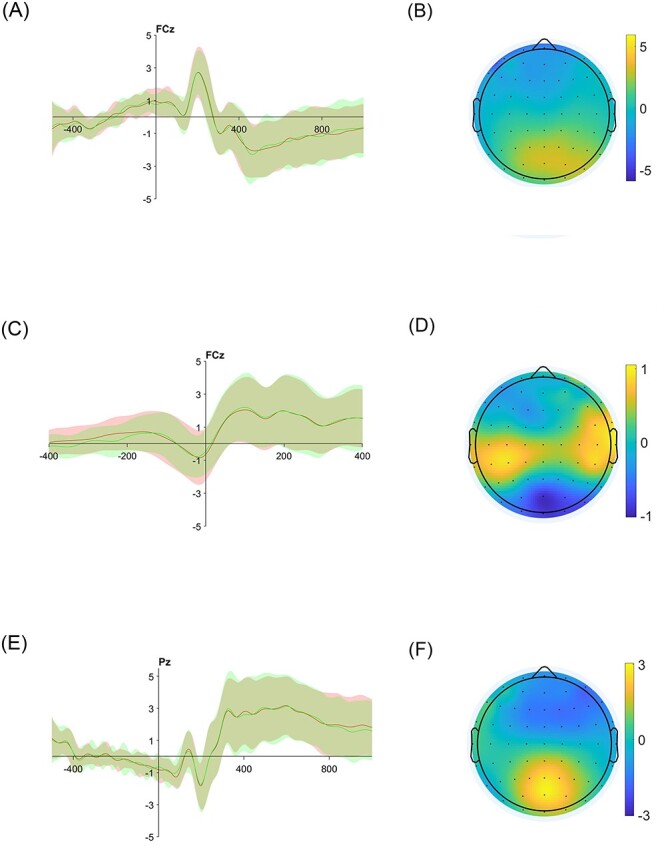
ERP results. (A) Stimulus-locked ERP waveforms in the FCz electrode (shaded area represents the point-by-point standard error). (B) The scalp distribution for the possible N2 component (300–330 ms following stimulus onset). (C) Response-locked ERP waveforms in the FCz electrode. (D) The scalp distribution for the possible CRN component (−50 to 50 ms around response onset). (E) Stimulus-locked ERP waveforms in the Pz electrode. (F) The scalp distribution for the possible LPC component, 650–750 ms following stimulus onset.

For the time–frequency decomposition (needed for the analysis of midfrontal theta power), we created long (5000 ms) stimulus-locked epochs starting 1000 ms before stimulus onset to avoid potential time–frequency decomposition edge artifacts. The time–frequency decomposition was done via EEGLAB (Delorme and Makeig, 2004). We extracted 38 frequencies in logarithmically spaced steps from 3 to 40 Hz. The time–frequency decomposition was based on Morlet wavelets with the number of cycles increasing logarithmically from three cycles at 3 Hz to eight cycles at 40 Hz to provide a continuous measure of the amplitude of a frequency component. We normalized the time–frequency data of each condition to a baseline of all time points before stimulus onset by conversion to a decibel scale, 10 log10 [power(*t*)/power(baseline)], allowing for a direct comparison of effects across frequency bands. To measure the overall midfrontal theta power, the sum of the power in the time interval between 0 and 3000 ms at 3.2–7.7 Hz (Lin *et al.*, 2018) was extracted. We chose 0–3000 ms as our time interval to avoid leakage of any spectral components that were sourced in response execution, as the RT for 75% of the trials exceeded 3000 ms.

## Results

### Behavioral results

A paired-samples one-tailed *t*-test, comparing RTs in AV-AV *vs* AP-AP trials, revealed a significant difference such that AV-AV conflicts (*M* = 5774 ms and s.d. = 1592) took longer to resolve compared to AP-AP conflicts (*M* = 4601 ms and s.d. = 1146), *t*(28) = 7.79, *P* < 0.001, *d* = 1.44. This result replicates previous findings (Arkoff, 1957; Atthowe, 1960; Minor *et al.*, 1968; Terry, 2010; Boyd *et al.*, 2011; Heitmann and Deutsch, 2019).

### Midfrontal theta power in AP-AP *vs* AV-AV conflicts

In our main analysis, we tested whether AP-AP conflicts and AV-AV conflicts differ in midfrontal theta power. We focused on the FCz electrode following previous studies that observed the strongest conflict-related theta effects on this electrode (Cavanagh *et al.*, 2012; Lin *et al.*, 2018). The time–frequency decomposition of electrode FCz for the two types of conflict is plotted in Figure 2A. We observed an overall increase in stimulus-locked theta power (3.2–7.7 Hz) after conflict presentation. A paired-samples one-tailed *t-*test, comparing overall theta power in the time window of 0–3000 ms following stimulus onset, revealed the predicted significant difference between the two conflict types such that theta power was higher in AV-AV conflicts compared to AP-AP ones, *t*(28) = 2.34, *P* = 0.013, *d* = 0.46 (Figure 2C).^3^

We next investigated the temporal dynamics of the differences in midfrontal theta power between the two conflict types. Specifically, we were interested in whether the difference between the conditions is sourced in a specific time point or rather reflects a prolonged effect through the processing of the conflict. To answer this question, we compared the time series of midfrontal theta power in AV-AV and AP-AP trials using a non-parametric cluster-based permutation test (Maris and Oostenveld, 2007). This method allows us to describe the time window in which theta power in AV-AV trials was higher than midfrontal theta power in AP-AP trials. The results of the cluster permutation test are plotted in Figure 2D. The test revealed a significant cluster in which theta power was higher in AV-AV trials than in AP-AP trials (cluster *t*-value_sum_ = 200.37, *P*_corr_ = 0.016). This effect corresponded to a time window ranging over a prolonged period, beginning at ∼600–650 ms following stimulus onset (note that the mean RT was 5203 ms) and lasting up until ∼3000 ms following the presentation of the conflict.

### Predicting decision times from midfrontal theta on a single-trial basis

We next sought to investigate the relationship between midfrontal theta, quantified on a single-trial basis, and decision times. To the extent that midfrontal theta is a mechanism for the recruitment of cognitive control needed to resolve conflicts (Cavanagh and Frank, 2014), trials with higher midfrontal theta power should result in more controlled behavior, i.e. shorter decision (conflict resolution) times (see the Discussion section for a more elaborate and nuanced account of this prediction). We were additionally interested in whether the relationship between midfrontal theta and decision times differs between conflict types. Thus, we assigned RT to linear mixed models, using the R package *lme4* (Version 1.1–27.1; Bates *et al.*, 2011) and the R package *lmerTest* (Kuznetsova *et al.*, 2017) with theta and conflict type as predictors and with random intercepts for participants. Theta power and RT values were centered before assigning them to the model. As hypothesized, we found a significant main effect for theta power, indicating that theta power negatively predicts RT, *B = *−147.05, *SE* = 23.85, *t* = −6.16, *P* < 0.001. In addition, we found an interaction between conflict type and theta power, such that the relationship between theta power and RT was stronger in AV-AV trials than in AP-AP trials, *B = *−56.89, *SE* = 22.05, *t* = −2.58, *P* = 0.009 (Figure 3A). Separate analysis for each conflict type indicated that theta power was negatively related to RT in both types of conflict, AV-AV: *B = *−205.32, *SE* = 36.44, *t* = −5.63, *P* < 0.001; AP-AP: *B = *−93.55, *SE* = 30.52, *t* = −3.06, *P* = 0.002.

### Conflict-related ERPs

The ERP waveforms in FCz electrode (used to quantify the N2 and CRN components), for stimulus-locked averaging and the response-locked averaging, are plotted in Figures 4A and 4C, respectively. The ERP waveform in Pz electrode (used to quantify the LPC component) is plotted in Figure 4E. We quantified the N2 component as the negative peak occurring 250–350 ms after stimulus onset in the FCz electrode and extracted peak amplitude and peak latency, consistent with the N2 component characteristics (Larson *et al.*, 2014). The scalp distribution for this N2 component is plotted in Figure 4B. As hypothesized, a paired-samples *t*-test did not reveal a difference between the conflict types in either peak amplitude (AV-AV: *M* = −1.38 μV, s.d. = 1.33, AP-AP: *M* = −1.47 μV, s.d. = 1.31; *t*(28) = 0.68, *P* = 0.501, *d* = 0.12) or peak latency (AV-AV: *M* = 311 ms, s.d. = 20, AP-AP: *M* = 313 ms, s.d. = 23; *t*(28) = 0.95, *P* = 0.352, *d* = 0.17). These null results are consistent with those of Lin *et al.* (2018); for an elaboration on this point, see the Introduction and Discussion sections.

Next, we looked at the CRN ERP component. In both conditions, we observed a negative peak over midfrontal scalp electrodes ∼6 ms before stimulus onset, consistent with the CRN component characteristics (Nakao *et al.*, 2010). We quantified CRN as a negative peak from 50 ms before response to 50 ms after the response and extracted peak amplitude and peak latency. A paired-samples *t*-test did not reveal a difference between the conflict types in either peak amplitude (AV-AV: *M* = −0.98 μV, s.d. = 1.23, AP-AP: *M* = −0.95 μV, s.d. = 1.08; *t*(28) = 0.27, *P* = 0.792, *d* = 0.04) or peak latency (AV-AV: *M* = −13 ms, s.d. = 15 , AP-AP: *M* = −18 ms, s.d. = 20; *t*(28) = 1.54, *P* = 0.134, *d* = 0.28).

We then looked at the LPC component. We quantified LPC as the mean amplitude 650–750 ms after stimulus presentation. A paired-samples *t*-test did not reveal any difference between the conditions. AV-AV: *M* = 2.56 μV, s.d. = 1.78, AP-AP: *M* = 2.62 μV, s.d. = 1.81; *t*(28) = 0.37, *P* = 0.716, *d* = 0.06.

## Discussion

In the present research, we investigated the neural underpinnings of the difference between AP-AP and AV-AV conflicts. Behaviorally, we replicated the results from previous studies and found that decision times were substantially longer for AV-AV conflicts than for AP-AP conflicts. With respect to the goal of the present research, we found that midfrontal theta power, a neural conflict marker, was higher in AV-AV conflicts than in AP-AP conflicts, for a prolonged period starting whole seconds before the actual motor response. We further found that higher levels of midfrontal theta were associated with shorter decision times on a single-trial basis, indicating successful recruitment of control, and that this effect was stronger in AV-AV conflicts than in AP-AP ones. Taken together, our results show that AP-AP and AV-AV conflicts are distinguishable on the neural level and at a much earlier stage than looking at their decision times would allow for. Our results thus establish midfrontal theta as a measure of the degree of conflict in motivational conflicts and are a neural documentation (see also Di Domenico *et al.*, 2016) of this seminal theory (Lewin, 1931; Miller, 1944). Moreover, our findings support the possibility that theta is a sensitive marker of continuous degrees of conflict (Lin *et al.*, 2018; Senftleben and Scherbaum, 2021).

There is an ongoing debate in the literature regarding the specific role of midfrontal theta oscillations in conflict processing. One line of research suggests that the anterior cingulate cortex, along with its theta oscillations, serves as a conflict detection system, while other cortical areas are responsible for conflict resolution (Botvinick *et al.*, 2004; Kaiser and Schütz-Bosbach, 2019). Other researchers propose that midfrontal theta is also involved in ongoing conflict resolution by facilitating alternative courses of action through control mechanisms (Cavanagh and Frank, 2014; Töllner *et al.*, 2017; Kaiser and Schütz-Bosbach, 2021) or by mediating the deliberation between response options (Senftleben and Scherbaum, 2021). Our study on midfrontal theta in motivational conflicts can provide a unique perspective regarding this debate. Previous studies were not interested in investigating theta in the context of prolonged decisions and thus were focused on a rather narrow time window for theta effects (e.g. Cavanagh *et al.*, 2012; Töllner *et al.*, 2017; Senftleben and Scherbaum, 2021). We employed an experimental task with prolonged decision times and observed a sustained effect of midfrontal theta, starting several seconds before the motor response and lasting ∼2.5 s. The finding that midfrontal theta effects persist throughout the extended duration of the conflict task provides support for the notion that these oscillations reflect continuous deliberation between response options, extending beyond the mere moment of conflict detection into conflict resolution.

We found that when resolving motivational conflicts, higher midfrontal theta power was associated with shorter decision (conflict resolution) times on a single-trial basis. Contrastingly, previous research that examined SRC tasks has found decision times to be longer in trials with high midfrontal theta power (Cohen and Cavanagh, 2011; Kaiser and Schütz-Bosbach, 2021). We find this discrepancy informative, as we believe that it highlights the flexible and adaptive nature of cognitive control in goal attainment. A primary goal in SRC tasks is selecting the correct response. Thus, when confronted with conflict, control mechanisms are necessary to delay an immediate (incorrect) response and enable the consideration of the alternative (Kaiser and Schütz-Bosbach, 2021; Kaiser *et al.*, 2022). Consequently, trials with higher theta power (indicating increased control implementation) are expected to result in longer decision times. Conversely, in motivational conflicts, where there are no objectively correct responses, a primary goal is simply to make a decision. Previous research has shown that when facing high-conflict decisions, individuals tend to defer the decision rather than to make it, which may in turn result in negative consequences (e.g. Murray, 1975; Dhar and Sherman, 1996; Chernev, 2004). In a choice setting where there is no correct decision, one could presumably deliberate endlessly. In such settings, control mechanisms may be required to facilitate making the decision. Thus, trials with higher theta power (indicating increased control implementation) are expected to result in shorter decision times, which is consistent with our findings. This explanation gains further support from our finding that the relationship between theta and decision times was stronger in AV-AV decisions than in AP-AP ones. AV-AV decisions elicit a greater tendency for deferral (Miller, 1944; Barker, 1946; Murray, 1975; Dhar and Sherman, 1996; Chernev, 2004), thereby making control implementation (as reflected in theta power) more critical for their resolution. Overall, our findings attest to the flexible nature of control in different types of conflict tasks, highlighting the complex interplay between cognitive control and decision-making processes.^4^

With regard to ERPs, all the known conflict-related components that we analyzed—CRN, N2 and the LPC—did not reflect the difference between AP-AP and AV-AV conflicts. These results were in line with our hypothesis. There are two factors that may account for these findings. First, as is typically the case for motivational conflicts (Arkoff, 1957; Minor *et al.*, 1968; Terry, 2010; Heitmann and Deutsch, 2019), in the present study as well, conflict resolution times were relatively long (mean = 5203 ms). The prolonged decision times might increase the temporal variability in the neural processes that underlie conflict resolution, which in turn leads to the masking of ERP components (Luck, 2014). This explanation is in line with previous studies that utilized conflict tasks with extended decision times and did not find conflict-related ERP effects (Stahl *et al.*, 2020; Givon *et al.*, 2022). Second, unlike the case of SRC tasks where ERP components have been established as markers of conflict (Larson *et al.*, 2014), the distinction between AP-AP and AV-AV conflicts lies in the degree of conflict rather than in its mere presence or absence. It is possible that conflict-related ERP components are more sensitive to the presence or absence of conflict rather than to continuous differences in its degree. This explanation is supported by the findings by Lin *et al.* (2018) who investigated a continuous intertemporal choice task and did not find N2 differentiation between conflicts of different degrees. The CRN component has been shown to differentiate between conflicts of varying degrees (Di Domenico *et al.*, 2016). However, it is worth noting that this effect size was relatively small, which could explain its absence in our data. Further research is thus needed to determine the exact nature of ERP effects in continuous, subjective, and prolonged decision tasks.

One could argue that the effect we observed in the present study for midfrontal theta was a result of the negative *vs* positive valence of the words presented on the screen rather than an enhanced conflict in AV-AV *vs* AP-AP decisions. However, we believe that the observed theta effect was sourced, at least to some extent, from the differences in the degree of conflict between the conditions. Midfrontal theta oscillations are widely known as an index of conflict processing and control recruitment (Cavanagh *et al.*, 2012; Cohen and Donner, 2013; Frank *et al.*, 2015; Lin *et al.*, 2018; Hsieh *et al.*, 2020; Senftleben and Scherbaum, 2021; Kaiser *et al.*, 2022) but are not considered to be an index of negative valance *per se*. Previous studies (e.g. Cohen *et al.*, 2007; van Driel *et al.*, 2012; Kaiser *et al.*, 2021) have indeed found increased theta power following negative feedback, but it was interpreted as reflecting the implementation of control to facilitate behavioral adjustments rather than the mere processing of negative valence. Additionally, our findings revealed that higher theta oscillations were predictive of shorter decision times, which is a well-established behavioral measure of successful control recruitment (Lowe and Mitterer, 1982; Braem *et al.*, 2019; Kaiser *et al.*, 2022). This finding supports the notion that theta serves as an index of conflict and the subsequent resolution process rather than being solely indicative of valence.

The present work relates to a growing body of research focusing on the neural mechanisms of various aspects of subjective decision-making, such as evidence accumulation (Summerfield and Tsetsos, 2012; Polanía *et al.*, 2014; Pisauro *et al.*, 2017), representation of reward for each of the choice options (Balleine *et al.*, 2007; Rushworth *et al.*, 2009; Carlson *et al.*, 2011) and the inherent reward of the choice itself (Leotti and Delgado, 2011). Here, we chose to focus on the concrete measures of conflict processing, as we aimed at understanding the neural mechanisms that make some conflicts more difficult to resolve than others. In future research, it would be interesting to combine our insights with insights into other aspects of decision-making to answer questions such as how the conflict type affects the value representation of different responses or what role theta oscillations play in the eventual decision.

To conclude, the theory of motivational conflicts was first proposed by Lewin (1931) and Miller (1944) some 90 years ago. Since then, the behavioral difficulty difference in resolving AP-AP and AV-AV conflicts has been documented by abundant research (Arkoff, 1957; Barker, 1946; Atthowe, 1960; Minor *et al.*, 1968; Murray, 1975; Terry, 2010; Boyd *et al.*, 2011; Perfecto *et al.*, 2017; Heitmann and Deutsch, 2019), yet the neural underpinnings of this difference remained less studied. Here, we tested the neural aspects of motivational conflict theory and found that midfrontal theta oscillations play an important role in tracking the difference between these two types of conflict. The implications of these results go beyond motivational conflicts, as they also contribute to the growing body of research that focuses on subjective and continuous decision-making processes.

## Supplementary Material

nsad038_Supp

## Data Availability

Data and analysis code for this article are available at the Open Science Framework at: https://osf.io/tezrj/.

## Footnotes

1 Including their data does not change the pattern of results.

2 The number of trials entering the analyses per participant and per condition can be found in the supplementary data.

3 To examine the specificity of the theta frequency band effect, we additionally analyzed alpha (8–12 Hz) and beta (12–30 Hz) frequency bands. We did not find significant differences between the two conflict types in either alpha or beta frequency bands. For the full analysis, see the supplementary data.

4 In the present paper, we used decision times as a behavioral indicator of the difference between the conflict types (as was done in abundant previous research). Future research could examine whether theta similarly predicts subjective difficulty ratings, which are another behavioral indicator of the difference between the two conflict types.

## Appendix

### Personal Characteristics Used as Stimuli in the Study

**Table:**

**Positive**	**Negative**
Interesting	Boring
Smart	Stupid
Confident	Insecure
Rich	Poor
Attractive	Ugly
Calm	Tense
Healthy	Sick

## References

[R1] Appelbaum L.G. , Meyerhoff K.L., Woldorff M.G. (2009). Priming and backward influences in the human brain: processing interactions during the Stroop interference effect. *Cerebral Cortex*, 19(11), 2508–21. 19321654 10.1093/cercor/bhp036 PMC2764508

[R2] Arkoff A. (1957). Resolution of approach-approach and avoidance-avoidance conflicts. *The Journal of Abnormal and Social Psychology*, 55(3), 402–4. 10.1037/h0043956 13474924

[R3] Atthowe J.M. Jr. (1960). Types of conflict and their resolution: a reinterpretation. *Journal of Experimental Psychology*, 59(1), 1–9. 13794998 10.1037/h0046912

[R4] Balleine B.W. , Delgado M.R., Hikosaka O. (2007). The role of the dorsal striatum in reward and decision-making. *Journal of Neuroscience*, 27(31), 8161–5. 17670959 10.1523/JNEUROSCI.1554-07.2007 PMC6673072

[R5] Barker R. (1946). An experimental study of the relationship between certainty of choice and the relative valence of the alternatives. *Journal of Personality*, 15, 41–52. 20288131 10.1111/j.1467-6494.1946.tb01049.x

[R6] Bates D. , Mächler M., Bolker B., Walker S. (2015). Fitting Linear Mixed-Effects Models Using lme4. *R Package Version*, 1(6).

[R7] Botvinick M.M. , Braver T.S., Barch D.M., Carter C.S., Cohen J.D. (2001). Conflict monitoring and cognitive control. *Psychological Review*, 108(3), 624–52. 11488380 10.1037/0033-295x.108.3.624

[R8] Botvinick M.M. , Cohen J.D., Carter C.S. (2004). Conflict monitoring and anterior cingulate cortex: an update. *Trends in Cognitive Sciences*, 8, 539–46. 15556023 10.1016/j.tics.2004.10.003

[R9] Boyd R.L. , Robinson M.D., Fetterman A.K. (2011). Miller (1944) revisited: movement times in relation to approach and avoidance conflicts. *Journal of Experimental Social Psychology*, 47(6), 1192–7.

[R10] Braem S. , Bugg J.M., Schmidt J.R., et al. (2019). Measuring adaptive control in conflict tasks. *Trends in Cognitive Sciences*, 23(9), 769–83. 31331794 10.1016/j.tics.2019.07.002 PMC6699878

[R11] Carlson J.M. , Foti D., Mujica-Parodi L.R., Harmon-Jones E., Hajcak G. (2011). Ventral striatal and medial prefrontal BOLD activation is correlated with reward-related electrocortical activity: a combined ERP and fMRI study. *NeuroImage*, 57(4), 1608–16. 21624476 10.1016/j.neuroimage.2011.05.037

[R12] Carter C.S. , van Veen V. (2007). Anterior cingulate cortex and conflict detection: an update of theory and data. *Cognitive, Affective, & Behavioral Neuroscience*, 7(4), 367–79. 10.3758/cabn.7.4.367 18189010

[R13] Cavanagh J.F. , Frank M.J. (2014). Frontal theta as a mechanism for cognitive control. *Trends in Cognitive Sciences*, 18(8), 414–21. 24835663 10.1016/j.tics.2014.04.012 PMC4112145

[R14] Cavanagh J.F. , Zambrano-Vazquez L., Allen J.J.B. (2012). Theta lingua franca: a common mid-frontal substrate for action monitoring processes. *Psychophysiology*, 49(2), 220–38. 22091878 10.1111/j.1469-8986.2011.01293.x PMC3262926

[R15] Chatterjee S. , Heath T.B. (1996). Conflict and loss aversion in multiattribute choice: the effects of trade-off size and reference dependence on decision difficulty. *Organizational Behavior and Human Decision Processes*, 67(2), 144–55.

[R16] Chernev A. (2004). Goal–attribute compatibility in consumer choice. *Journal of Consumer Psychology*, 14(1), 141–50.

[R17] Cohen M.X. (2014). A neural microcircuit for cognitive conflict detection and signaling. *Trends in Neurosciences*, 37(9), 480–90. 25034536 10.1016/j.tins.2014.06.004

[R18] Cohen M. , Cavanagh J.F. (2011). Single-trial regression elucidates the role of prefrontal theta oscillations in response conflict. *Frontiers in Psychology*, 2, 30. 10.3389/fpsyg.2011.00030 PMC3111011 21713190

[R19] Cohen M. , Donner T. (2013). Midfrontal conflict-related theta-band power reflects neural oscillations that predict behavior. *Journal of Neurophysiology*, 110, 2752–63. 24068756 10.1152/jn.00479.2013

[R20] Cohen M.X. , Elger C.E., Ranganath C. (2007). Reward expectation modulates feedback-related negativity and EEG spectra. *NeuroImage*, 35(2), 968–78. 17257860 10.1016/j.neuroimage.2006.11.056 PMC1868547

[R21] Delorme A. , Makeig S. (2004). EEGLAB: an open source toolbox for analysis of single-trial EEG dynamics including independent component analysis. *Journal of Neuroscience Methods*, 134(1), 9–21. 15102499 10.1016/j.jneumeth.2003.10.009

[R22] Dhar R. , Sherman S.J. (1996). The effect of common and unique features in consumer choice. *Journal of Consumer Research*, 23(3), 193–203.

[R23] Di Domenico S.I. , Fournier M.A., Ayaz H., Ruocco A.C. (2013). In search of integrative processes: basic psychological need satisfaction predicts medial prefrontal activation during decisional conflict. *Journal of Experimental Psychology: General*, 142, 967–78. 23067061 10.1037/a0030257

[R24] Di Domenico S.I. , Le A., Liu Y., Ayaz H., Fournier M.A. (2016). Basic psychological needs and neurophysiological responsiveness to decisional conflict: an event-related potential study of integrative self processes. *Cognitive, Affective, & Behavioral Neuroscience*, 16(5), 848–65. 10.3758/s13415-016-0436-1 27215614

[R25] Egner T. (2008). Multiple conflict-driven control mechanisms in the human brain. *Trends in Cognitive Sciences*, 12(10), 374–80. 18760657 10.1016/j.tics.2008.07.001

[R26] Eriksen B.A. , Eriksen C.W. (1974). Effects of noise letters upon the identification of a target letter in a nonsearch task. *Perception & Psychophysics*, 16(1), 143–9.

[R27] Frank M.J. , Gagne C., Nyhus E., et al. (2015). FMRI and EEG predictors of dynamic decision parameters during human reinforcement learning. *Journal of Neuroscience*, 35(2), 485–94. 25589744 10.1523/JNEUROSCI.2036-14.2015 PMC4293405

[R28] Givon E. , Udelsman-Danieli G., Almagor O., Fekete T., Shriki O., Meiran N. (2022). Can feelings “feel” wrong? Similarities between counter-normative emotion reports and perceptual errors. *Psychological Science*, 33(6), 948–56. 35503295 10.1177/09567976211063915

[R29] Heitmann C. , Deutsch R. (2019). Post-conflict speeding: evidence of sequential effects in motivational conflicts. *Journal of Experimental Psychology: Learning, Memory, and Cognition*, 45(3), 452–69. 30024262 10.1037/xlm0000585

[R30] Hsieh S.-S. , Chueh T.-Y., Morris T.P., et al. (2020). Greater childhood cardiorespiratory fitness is associated with better top-down cognitive control: a midfrontal theta oscillation study. *Psychophysiology*, 57(12), e13678. 10.1111/psyp.13678 32877574

[R31] Jung T.-P. , Makeig S., Westerfield M., Townsend J., Courchesne E., Sejnowski T.J. (2000). Removal of eye activity artifacts from visual event-related potentials in normal and clinical subjects. *Clinical Neurophysiology*, 111, 1745–58. 11018488 10.1016/s1388-2457(00)00386-2

[R32] Kaiser J. , Belenya R., Chung W.-Y., Gentsch A., Schütz-Bosbach S. (2021). Learning something new versus changing your ways: distinct effects on midfrontal oscillations and cardiac activity for learning and flexible adjustments. *NeuroImage*, 226, 117550. 10.1016/j.neuroimage.2020.117550 33186724

[R33] Kaiser J. , Iliopoulos P., Steinmassl K., Schütz-Bosbach S. (2022). Preparing for success: neural frontal theta and posterior alpha dynamics during action preparation predict flexible resolution of cognitive conflicts. *Journal of Cognitive Neuroscience*, 34(6), 1070–89. 35286387 10.1162/jocn_a_01846

[R34] Kaiser J. , Schütz-Bosbach S. (2019). Proactive control without midfrontal control signals? The role of midfrontal oscillations in preparatory conflict adjustments. *Biological Psychology*, 148, 107747. 10.1016/j.biopsycho.2019.107747 31470073

[R35] Kaiser J. , Schütz-Bosbach S. (2021). Motor interference, but not sensory interference, increases midfrontal theta activity and brain synchronization during reactive control. *The Journal of Neuroscience*, 41(8), 1788–801. 33441433 10.1523/JNEUROSCI.1682-20.2020 PMC8115891

[R36] Kornblum S. , Hasbroucq T., Osman A. (1990). Dimensional overlap: cognitive basis for stimulus-response compatibility—a model and taxonomy. *Psychological Review*, 97(2), 253. 10.1037/0033-295x.97.2.253 2186425

[R37] Kuznetsova A. , Brockhoff P.B., Christensen R.H.B. (2017). lmerTest package: tests in linear mixed effects models. *Journal of Statistical Software*, 82, 1–26.

[R38] Larson M.J. , Clawson A., Clayson P.E., South M. (2012). Cognitive control and conflict adaptation similarities in children and adults. *Developmental Neuropsychology*, 37(4), 343–57. 22612546 10.1080/87565641.2011.650337

[R39] Larson M.J. , Clayson P.E., Clawson A. (2014). Making sense of all the conflict: a theoretical review and critique of conflict-related ERPs. *International Journal of Psychophysiology*, 93(3), 283–97. 24950132 10.1016/j.ijpsycho.2014.06.007

[R40] Leotti L.A. , Delgado M.R. (2011). The inherent reward of choice. *Psychological Science*, 22(10), 1310–8. 21931157 10.1177/0956797611417005 PMC3391581

[R41] Lewin K. (1931). Environmental forces in child behavior and development. In: *A Handbook of Child Psychology*, Worcester, MA: Clark University Press. 94–127.

[R42] Lin H. , Saunders B., Hutcherson C.A., Inzlicht M. (2018). Midfrontal theta and pupil dilation parametrically track subjective conflict (but also surprise) during intertemporal choice. *NeuroImage*, 172, 838–52. 29107773 10.1016/j.neuroimage.2017.10.055

[R43] Lopez-Calderon J. , Luck S.J. (2014). ERPLAB: an open-source toolbox for the analysis of event-related potentials. *Frontiers in Human Neuroscience*, 8, 213. 10.3389/fnhum.2014.00213 PMC3995046 24782741

[R44] Lowe D.G. , Mitterer J.O. (1982). Selective and divided attention in a Stroop task. *Canadian Journal of Psychology/Revue Canadienne de Psychologie*, 36, 684–700. 10.1037/h0080661 7159848

[R45] Luck S.J. (2014). *An Introduction to the Event-related Potential Technique*, 2nd edn, Cambridge, Massachusetts: MIT Press.

[R46] MacDonald A.W. , Cohen J.D., Stenger V.A., Carter C.S. (2000). Dissociating the role of the dorsolateral prefrontal and anterior cingulate cortex in cognitive control. *Science*, 288(5472), 1835–8. 10846167 10.1126/science.288.5472.1835

[R47] Maris E. , Oostenveld R. (2007). Nonparametric statistical testing of EEG- and MEG-data. *Journal of Neuroscience Methods*, 164(1), 177–90. 17517438 10.1016/j.jneumeth.2007.03.024

[R48] McKay C.C. , van den Berg B., Woldorff M.G. (2017). Neural cascade of conflict processing: not just time-on-task. *Neuropsychologia*, 96, 184–91. 28017818 10.1016/j.neuropsychologia.2016.12.022 PMC5365079

[R49] Miller N.E. (1944). Experimental studies of conflict. In: *Personality and the Behavior Disorders*, Oxford, England: Ronald Press. 431–65.

[R50] Minor J. , Miller L., Ditrichs R. (1968). The effect of an undecided alternative on resolution of approach-approach and avoidance-avoidance conflict situations. *Psychonomic Science*, 12(8), 375–375.

[R51] Murray E.J. (1975). Resolution of complex decisional conflicts as a function of degree of avoidance. *Journal of Research in Personality*, 9(3), 177–90.

[R52] Nakao T. , Bai Y., Nashiwa H., Northoff G. (2013). Resting-state EEG power predicts conflict-related brain activity in internally guided but not in externally guided decision-making. *NeuroImage*, 66, 9–21. 23103687 10.1016/j.neuroimage.2012.10.034

[R53] Nakao T. , Mitsumoto M., Nashiwa H., et al. (2010). Self-knowledge reduces conflict by biasing one of plural possible answers. *Personality and Social Psychology Bulletin*, 36(4), 455–69. 20363902 10.1177/0146167210363403

[R54] Peirce J. , Gray J.R., Simpson S., et al. (2019). PsychoPy2: experiments in behavior made easy. *Behavior Research Methods*, 51(1), 195–203. 30734206 10.3758/s13428-018-01193-y PMC6420413

[R55] Perfecto H. , Galak J., Simmons J.P., Nelson L.D. (2017). Rejecting a bad option feels like choosing a good one. *Journal of Personality and Social Psychology*, 113(5), 659–70. 28737416 10.1037/pspa0000092

[R56] Pisauro M.A. , Fouragnan E., Retzler C., Philiastides M.G. (2017). Neural correlates of evidence accumulation during value-based decisions revealed via simultaneous EEG-fMRI. *Nature Communications*, 8(1), 15808. 10.1038/ncomms15808 PMC5472767 28598432

[R57] Polanía R. , Krajbich I., Grueschow M., Ruff C.C. (2014). Neural oscillations and synchronization differentially support evidence accumulation in perceptual and value-based decision making. *Neuron*, 82(3), 709–20. 24811387 10.1016/j.neuron.2014.03.014

[R58] Rushworth M.F. , Mars R.B., Summerfield C. (2009). General mechanisms for making decisions?. *Current Opinion in Neurobiology*, 19(1), 75–83. 19349160 10.1016/j.conb.2009.02.005

[R59] Senftleben U. , Scherbaum S. (2021). Mid-frontal theta during conflict in a value-based decision task. *Journal of Cognitive Neuroscience*, 33(10), 2109–31. 34407197 10.1162/jocn_a_01741

[R60] Stahl J. , Mattes A., Hundrieser M., et al. (2020). Neural correlates of error detection during complex response selection: introduction of a novel eight-alternative response task. *Biological Psychology*, 156, 107969. 10.1016/j.biopsycho.2020.107969 33058968

[R61] Stroop J.R. (1935). Studies of interference in serial verbal reactions. *Journal of Experimental Psychology*, 18(6), 643.

[R62] Summerfield C. , Tsetsos K. (2012). Building bridges between perceptual and economic decision-making: neural and computational mechanisms. *Frontiers in Neuroscience*, 6, 70. 10.3389/fnins.2012.00070 PMC3359443 22654730

[R63] Terry W.S. (2010). A demonstration of approach and avoidance conflicts. *Teaching of Psychology*, 37(2), 132–4.

[R64] Töllner T. , Wang Y., Makeig S., Müller H.J., Jung T.-P., Gramann K. (2017). Two independent frontal midline theta oscillations during conflict detection and adaptation in a Simon-type manual reaching task. *The Journal of Neuroscience*, 37(9), 2504–15. 28137968 10.1523/JNEUROSCI.1752-16.2017 PMC6596837

[R65] van Driel J. , Ridderinkhof K.R., Cohen M.X. (2012). Not all errors are alike: theta and alpha EEG dynamics relate to differences in error-processing dynamics. *The Journal of Neuroscience: The Official Journal of the Society for Neuroscience*, 32(47), 16795–806. 23175833 10.1523/JNEUROSCI.0802-12.2012 PMC6621791

[R66] Vanveen V. , Carter C. (2002). The anterior cingulate as a conflict monitor: FMRI and ERP studies. *Physiology & Behavior*, 77(4–5), 477–82. 12526986 10.1016/s0031-9384(02)00930-7

