# Supplementary material for: Midfrontal theta as an index of conflict strength in approach–approach vs avoidance–avoidance conflicts
Source: Soc Cogn Affect Neurosci. 2023 Jul 24;18(1):nsad038. doi: 10.1093/scan/nsad038 (PMC10411683; doi:10.1093/scan/nsad038)
Supplement: nsad038_Supp [file nsad038_supp.zip › scan-23-022-File002.docx]

# **Supplementary Information – *Midfrontal Theta as an Index of Conflict Strength in Approach-Approach vs. Avoidance-Avoidance Conflicts***

**Alpha and Beta frequency-bands analysis**^[[1]](#footnote-1)^

To examine the specificity of the effect presented in the main paper to the theta frequency-band, we analyzed the differences between AP-AP and AV-AV conflicts alpha (8–12 Hz) and beta (12–30Hz) frequency-bands. The time-frequency decomposition of electrode FCz for the two types of conflict is plotted in Figure 2a of the main paper. A paired-samples *t-*test, comparing overall alpha power in electrode FCz in the time window of 0–3000ms following stimulus onset, did not reveal a significant difference between the two conflict types *t*(28) = -0.68, *p* = .49, *d* = 0.12 (see supplementary Figure 1A). In addition, a paired-samples *t-*test, comparing overall beta power in electrode FCz in the time window of 0–3000ms following stimulus onset, did not reveal a significant difference between the two conflict types *t*(28) = 1.83, *p* = .07, *d* = 0.34 (see supplementary Figure 1B).

We additionally looked at alpha power at posterior regions. A paired -samples *t*-test, comparing overall alpha power in electrode Oz in the time window of 0–3000ms following stimulus onset did not reveal a significant difference between the conflict types *t*(28) = 1.31, *p* = 0.19, *d* = 0.24 (see supplementary Figure 1C).


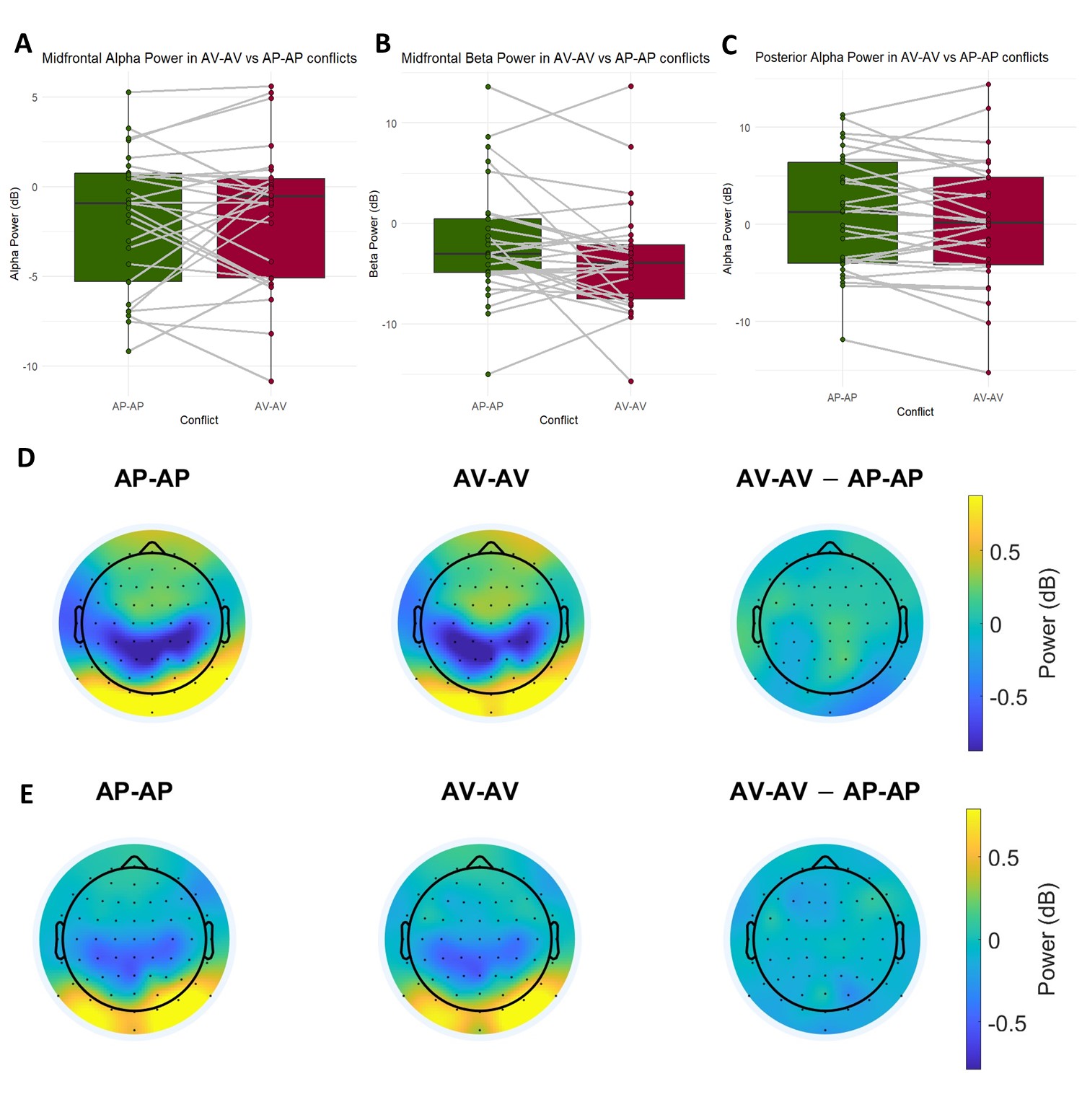


*Supplementary Figure 1*. **A-C,** Box plots of overall midfrontal alpha and beta power as a function of conflict type, measured in a time window of 0–3000ms following stimulus onset. The middle line represents the median and the whiskers represent 1.5 times the interquartile range. The pairs of connected dots (via the gray lines) represent the average of midfrontal alpha/beta power for each participant in each of the conflict conditions. **D,** Topographical plots for alpha power for each condition and for the average difference between conditions. **E,** Topographical plots for beta power for each condition and for the average difference between conditions.

| **Supplementary Table 1** | | | | |
| --- | --- | --- | --- | --- |
| *Trials included in the analysis^[[2]](#footnote-2)^* | | | | |
| Subject ID | Conflict | | Trials left after behavioral exclusion criteria | Trials left after EEG artifact rejection |
| 3 | AP-AP | 144 | | 107 |
| 3 | AV-AV | 142 | | 99 |
| 4 | AP-AP | 133 | | 115 |
| 4 | AV-AV | 133 | | 112 |
| 5 | AP-AP | 145 | | 114 |
| 5 | AV-AV | 144 | | 104 |
| 6 | AP-AP | 147 | | 114 |
| 6 | AV-AV | 143 | | 109 |
| 7 | AP-AP | 146 | | 114 |
| 7 | AV-AV | 144 | | 113 |
| 9 | AP-AP | 143 | | 139 |
| 9 | AV-AV | 146 | | 142 |
| 10 | AP-AP | 144 | | 142 |
| 10 | AV-AV | 137 | | 132 |
| 11 | AP-AP | 146 | | 123 |
| 11 | AV-AV | 143 | | 133 |
| 13 | AP-AP | 143 | | 102 |
| 13 | AV-AV | 142 | | 104 |
| 14 | AP-AP | 124 | | 115 |
| 14 | AV-AV | 123 | | 114 |
| 15 | AP-AP | 136 | | 112 |
| 15 | AV-AV | 135 | | 109 |
| 16 | AP-AP | 146 | | 121 |
| 16 | AV-AV | 145 | | 123 |
| 17 | AP-AP | 141 | | 100 |
| 17 | AV-AV | 139 | | 104 |
| 18 | AP-AP | 147 | | 122 |
| 18 | AV-AV | 143 | | 109 |
| 19 | AP-AP | 146 | | 131 |
| 19 | AV-AV | 143 | | 128 |
| 20 | AP-AP | 140 | | 129 |
| 20 | AV-AV | 133 | | 115 |
| 21 | AP-AP | 145 | | 117 |
| 21 | AV-AV | 146 | | 119 |
| 23 | AP-AP | 147 | | 142 |
| 23 | AV-AV | 144 | | 141 |
| 24 | AP-AP | 147 | | 114 |
| 24 | AV-AV | 142 | | 106 |
| 25 | AP-AP | 145 | | 113 |
| 25 | AV-AV | 141 | | 102 |
| 26 | AP-AP | 144 | | 130 |
| 26 | AV-AV | 143 | | 131 |
| 27 | AP-AP | 145 | | 106 |
| 27 | AV-AV | 144 | | 114 |
| 29 | AP-AP | 146 | | 137 |
| 29 | AV-AV | 143 | | 135 |
| 30 | AP-AP | 145 | | 135 |
| 30 | AV-AV | 145 | | 138 |
| 31 | AP-AP | 146 | | 126 |
| 31 | AV-AV | 143 | | 117 |
| 32 | AP-AP | 143 | | 115 |
| 32 | AV-AV | 143 | | 120 |
| 33 | AP-AP | 145 | | 134 |
| 33 | AV-AV | 144 | | 133 |
| 34 | AP-AP | 145 | | 128 |
| 34 | AV-AV | 144 | | 118 |
| 35 | AP-AP | 145 | | 103 |
| 35 | AV-AV | 141 | | 114 |

1. The following analyses were done per the editor’s and reviewers’ suggestions during the review process. [↑](#footnote-ref-1)
2. Note that in order to use the same pool of trials for all analyses, the trials rejected via the artifact rejection process of the ERP analysis were also rejected from the time frequency decomposition, and from the mixed-effect model analysis, predicting decision times from midfrontal theta on a single-trial basis. However, all of the results of all mfTheta-based analyses remains the same when including these trials. [↑](#footnote-ref-2)
