# Supplementary material for: Midfrontal theta as an index of conflict strength in approach–approach vs avoidance–avoidance conflicts
Source: Soc Cogn Affect Neurosci. 2023 Jul 24;18(1):nsad038. doi: 10.1093/scan/nsad038 (PMC10411683; doi:10.1093/scan/nsad038)
Supplement: nsad038_Supp [file nsad038_supp.zip › Appendix.docx]

## Appendix

*Personal characteristics* *used as* *stimuli in the study*

| Positive | Negative |
| --- | --- |
| Interesting | Boring |
| Smart | Stupid |
| Confident | Insecure |
| Rich | Poor |
| Attractive | Ugly |
| Calm | Tense |
| Healthy | Sick |
