# Supplementary figures and images for: Midfrontal theta as an index of conflict strength in approach–approach vs avoidance–avoidance conflicts
Source: Soc Cogn Affect Neurosci. 2023 Jul 24;18(1):nsad038. doi: 10.1093/scan/nsad038 (PMC10411683; doi:10.1093/scan/nsad038)

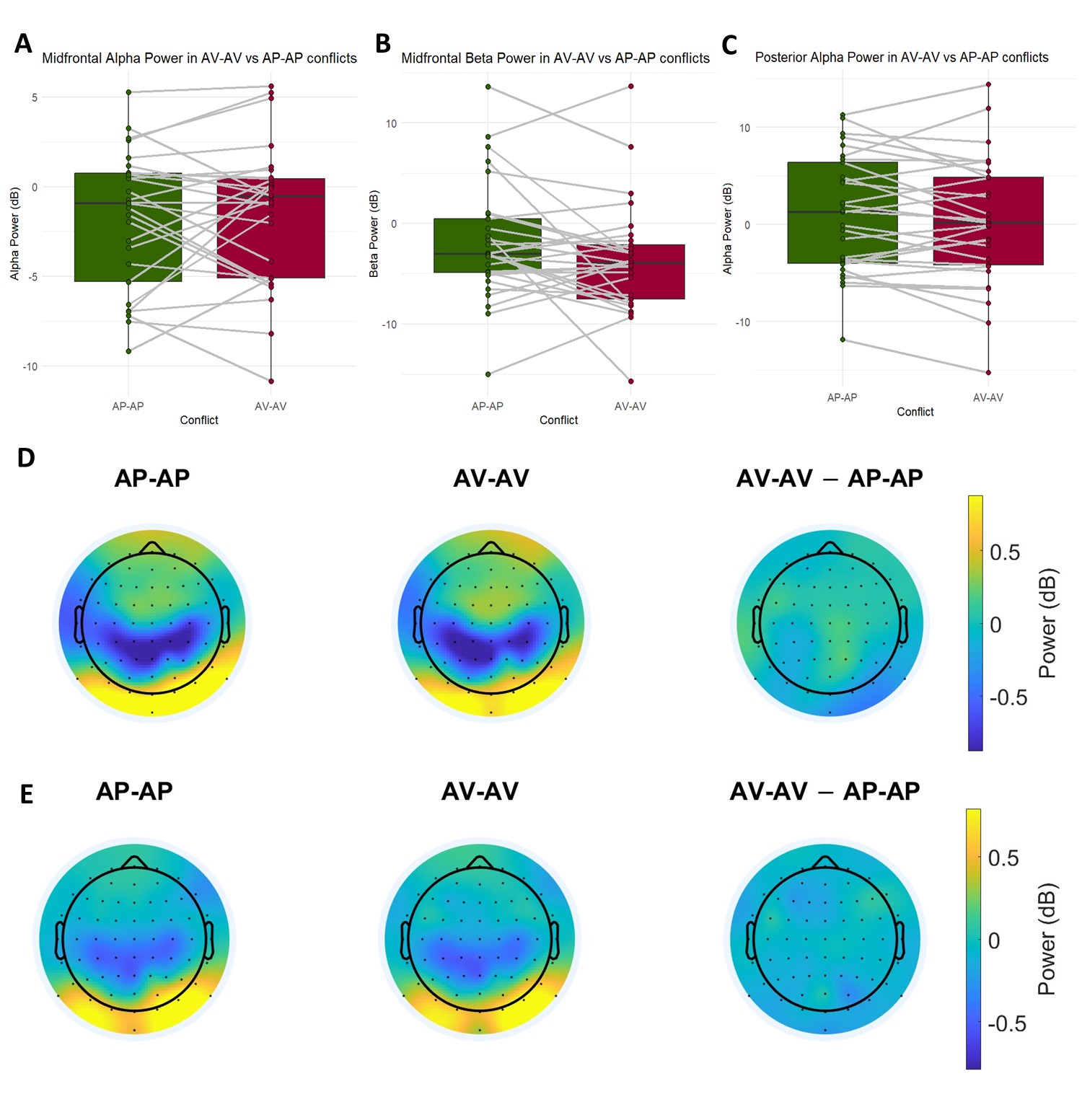

Supplement: nsad038_Supp [file nsad038_supp.zip › scan-23-022-File016.jpg]
